# Rare variant of the epigenetic regulator SMCHD1 in a patient with pituitary hormone deficiency

**DOI:** 10.1038/s41598-020-67715-x

**Published:** 2020-07-03

**Authors:** Kenichi Kinjo, Keisuke Nagasaki, Koji Muroya, Erina Suzuki, Keisuke Ishiwata, Kazuhiko Nakabayashi, Atsushi Hattori, Koji Nagao, Ryu-Suke Nozawa, Chikashi Obuse, Kenji Miyado, Tsutomu Ogata, Maki Fukami, Mami Miyado

**Affiliations:** 1 Department of Molecular Endocrinology, National Research Institute for Child Health and Development, Tokyo, Japan; 2 Department of Pediatrics, Hamamatsu University School of Medicine, Hamamatsu, Japan; 3 Department of Homeostatic Regulation and Development, Niigata University Graduate School of Medical and Dental Sciences, Niigata, Japan; 4 Department of Endocrinology and Metabolism, Kanagawa Children’s Medical Center, Yokohama, Japan; 5 Department of Maternal-Fetal Biology, National Research Institute for Child Health and Development, Tokyo, Japan; 6 Department of Advanced Pediatric Medicine, Tohoku University School of Medicine, Tokyo, Japan; 7 Department of Biological Sciences, Graduate School of Science, Osaka University, Osaka, Japan; 8 Department of Reproductive Biology, National Research Institute for Child Health and Development, Tokyo, Japan; 9 Present Address: Division of Experimental Pathology, The Cancer Institute of JFCR, Tokyo, Japan

**Keywords:** Genetics, Diseases, Endocrinology, Medical research, Molecular medicine

## Abstract

Isolated hypogonadotropic hypogonadism (IHH), combined pituitary hormone deficiency (CPHD), and septo-optic dysplasia (SOD) constitute a disease spectrum whose etiology remains largely unknown. This study aimed to clarify whether mutations in *SMCHD1*, an epigenetic regulator gene, might underlie this disease spectrum. *SMCHD1* is a causative gene for Bosma arhinia microphthalmia syndrome characterized by arhinia, microphthalmia and IHH. We performed mutation screening of *SMCHD1* in patients with etiology-unknown IHH (n = 31) or CPHD (n = 43, 19 of whom also satisfied the SOD diagnostic criteria). Rare variants were subjected to in silico analyses and classified according to the American College of Medical Genetics and Genomics guidelines. Consequently, a rare likely pathogenic variant, p.Asp398Asn, was identified in one patient. The patient with p.Asp398Asn exhibited CPHD, optic nerve hypoplasia, and a thin retinal nerve fiber layer, and therefore satisfied the criteria of SOD. This patient showed a relatively low DNA methylation level of the 52 SMCHD1-target CpG sites at the *D4Z4* locus. Exome sequencing for the patient excluded additional variants in other IHH/CPHD-causative genes. In vitro assays suggested functional impairment of the p.Asp398Asn variant. These results provide the first indication that *SMCHD1* mutations represent a rare genetic cause of the HH-related disease spectrum.

## Introduction

Hypogonadotropic hypogonadism (HH) is a multifactorial disorder that occurs either as an isolated hormonal abnormality (isolated HH, IHH) or in combination with other pituitary hormone deficiencies (combined pituitary hormone deficiency, CPHD)^1,2^. HH is frequently accompanied by craniofacial and neurological abnormalities, such as microphthalmia, anosmia, and septum pellucidum/corpus callosum hypoplasia^2–6^. The current understanding is that IHH and CPHD belong to a disease spectrum, which includes septo-optic dysplasia (SOD) and holoprosencephaly at the most severe end and IHH at the mildest end^1,7^. This disease spectrum results from defective organogenesis from the cranial placodes^1,8^. To date, more than 40 genes have been implicated in this disease spectrum^1,9^. However, mutations in these genes account for only about 50% of IHH cases and less than 20% of CPHD cases, indicating that other causative genes remain to be identified^1,9^.

*SMCHD1* encodes an epigenetic regulator that controls DNA methylation of multiple genomic loci^10–12^. Previous studies have shown that SMCHD1 is involved in the regulation of several monoallelically expressed genes^11,13^, and is particularly enriched in the nuclear territory of the inactive X chromosome (Xi)^14, 15^. Yet, the precise function of this protein remains to be clarified. Recently, heterozygous *SMCHD1* mutations were identified in patients with Bosma arhinia microphthalmia syndrome (BAMS), an extremely rare syndrome whose triad is the absence of the nose, microphthalmia, and IHH^16–18^. In addition, *SMCHD1* mutations are known to cause facioscapulohumeral muscular dystrophy type 2 (FSHD2), particularly when the mutations co-occurred with disease-susceptible alleles at the *D4Z4* locus^19,20^. BAMS/FSHD2 patients with *SMCHD1* mutations frequently exhibit DNA hypomethylation in the *DUX4* promoter region at the *D4Z4* locus, possibly reflecting impaired regulatory activity of the mutant SMCHD1 proteins^16,19^. Previous studies revealed that BAMS-causative *SMCHD1* mutations consist solely of missense substitutions within or very close to the GHKL ATPase domain, while FSHD2-causative mutations include several missense, nonsense, and frameshift variants widely distributed in the 48 coding exons^16–18^. It has been proposed that gain-of-function and loss-of-function mutations in *SMCHD1* result in BAMS and FSHD2, respectively, although four mutations have been associated with both conditions^16,18,21, 22^. More than 40 sporadic and familial cases with BAMS due to *SMCHD1* mutations have been reported to date^16–18^. Clinical analysis of the familial cases suggested variable expressivity of the BAMS triad^16^. However, given that previous molecular analyses of *SMCHD1* primarily focused on individuals with facial anomalies or muscular dystrophy, further studies are needed to clarify the phenotypic variations of *SMCHD1* abnormalities. In particular, it remains to be elucidated whether *SMCHD1* mutations can underlie IHH or CPHD cases without nasal malformations. To address this issue, we performed mutation screening of *SMCHD1* in 74 patients with HH-related disorders.

## Results

### Mutation screening of *SMCHD1* for 74 patients with HH-related disorders

The study was approved by the Institutional Review Board Committee at the National Center for Child and Development and performed after obtaining informed consent from the participants or their parents. We performed mutation screening of *SMCHD1* in 74 patients, i.e., 31 patients clinically diagnosed with IHH and 43 patients with CPHD. Nineteen of the CPHD patients also satisfied the diagnostic criteria of SOD^23^. All 74 patients had normal nasal structures and harbored no pathogenic variants in any of the known causative genes of IHH or CPHD.

All 48 coding exons of *SMCHD1* and their flanking intronic regions were amplified and subjected to next generation sequencing. The functional consequence of each variant was assessed by in silico analyses. The variants were classified according to the American College of Medical Genetics and Genomics and the Association for Molecular Pathology (ACMG/AMP) guidelines for interpretation of sequence variants^24^.

**Figure 1:**
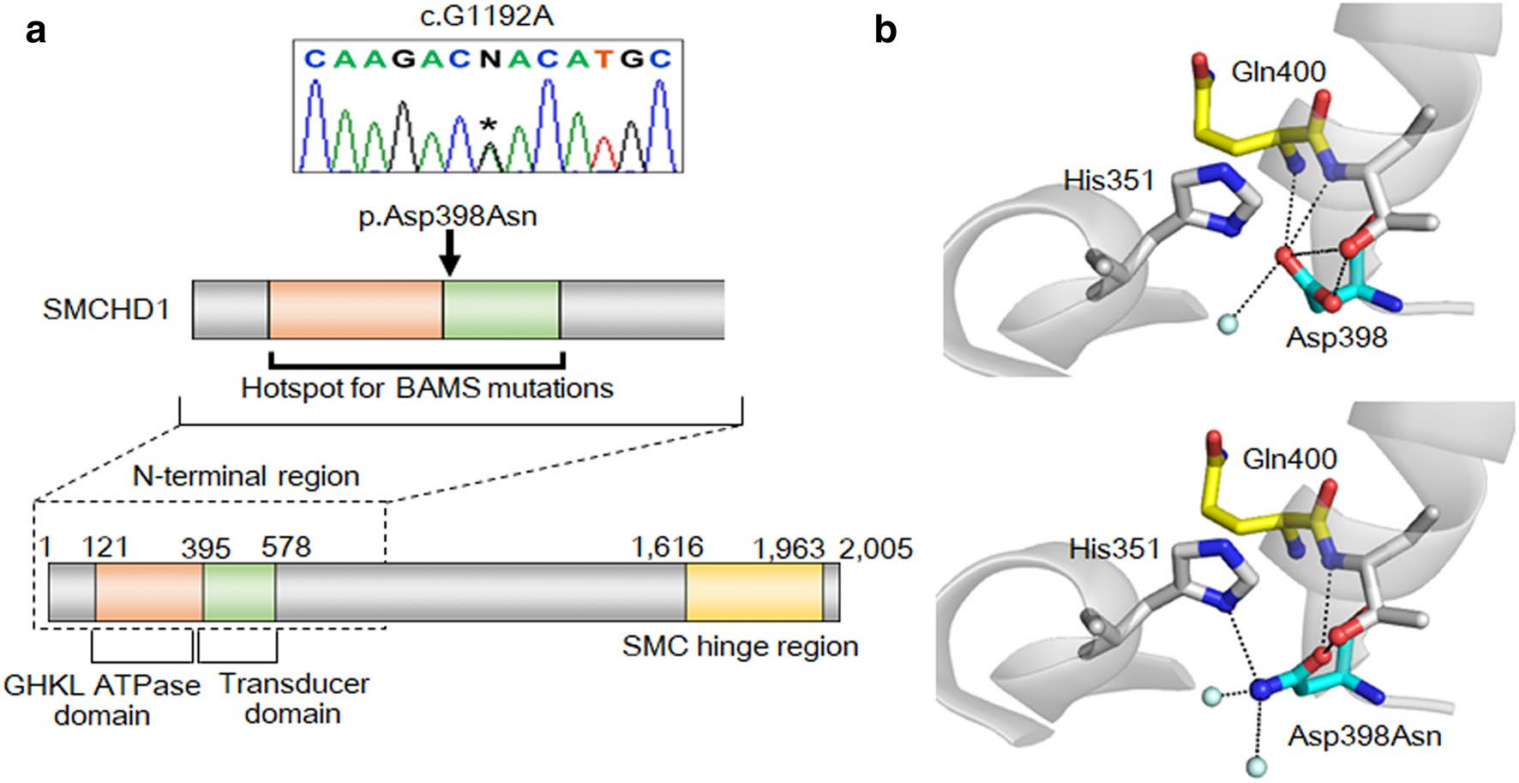
The p.Asp398Asn variant of SMCHD1. (**a**) The genomic position of the c.G1192A variant. The asterisk in the chromatogram denotes the mutated nucleotide. This variant resided in exon 10, within the hotspot of Bosma arhinia microphthalmia syndrome (BAMS) mutations^18^. The orange, green, and yellow indicate the GHKL ATPase domain, the transducer domain, and SMC hinge region, respectively. (**b**) Protein structure prediction of the p.Asp398Asn variant. This variant was predicted to alter multiple hydrogen bonds.

Consequently, two rare heterozygous variants, namely, c.G1192A (p.Asp398Asn) and c.G54C (p.Glu18Asp), were identified in patients 1 and 2, respectively (Table 1). Patients 1 and 2 were from the CPHD and IHH groups, respectively. Of the two variants, p.Asp398Asn in patient 1 was scored as deleterious by all of the five in silico programs used in this study. This variant has not been submitted to the ClinVar database. The variant resides in exon 10 within the hotspot for BAMS-causative mutations^18^, and affects a conserved amino acid adjacent to the ATPase domain (Fig. 1a). In silico protein structural analysis suggested that this substitution creates a new hydrogen bond between the 398th and 351st residues and disrupts a hydrogen bond between the 398th and 400th residues (Fig. 1b). The p.Asp398Asn variant was ranked as likely pathogenic according to the ACMG/AMP guidelines (Table 1). The variant was confirmed by Sanger sequencing (Fig. 1a). Although we attempted to obtain DNA samples from the patient’s parents, such samples were unavailable.

**Table 1:** Characteristics of two rare *SMCHD1* variants.

**Patient**	**Pheno-type**	**Variant**		**ClinVar**^**a**^	**dbSNP**^**b**^	**Allele frequency**			**In silico analysis**					**ACMG/AMP guideline**
		**cDNA**	**Protein**			**1000G**^**c**^	**gnomAD**^**d**^	**HGVD**^**e**^	**PolyPhen-2**^**f**^	**SIFT**^**g**^	**CADD**^**h**^	**M-CAP**^**i**^	**Mutation-Taster**^**j**^	
1	CPHD	c.G1192A	p.Asp398Asn	No data	No data	No data	No data	No data	**1.000**	**0.000**	**26.2**	**0.163**	**Disease causing**	Likely pathogenic
2	IHH	c.G54C	p.Glu18Asp	Likely benign	rs779165187	No data	20/7,642	5/2,402	0.004	0.246	9.9	**0.087**	Polymorphism	Likely benign

*ACMG/AMP* the American College of Medical Genetics and Genomics and the Association for Molecular Pathology, *CPHD* combined pituitary hormone deficiency, *IHH* Isolated hypogonadotropic hypogonadism. Scores indicative of a damaging variant are boldfaced.

^a^ClinVar (https://www.ncbi.nlm.nih.gov/clinvar/).

^b^dbSNP (https://www.ncbi.nlm.nih.gov/snp/).

^c^The 1000 Genome Database (https://www.internationalgenome.org/1000-genomes-browsers/).

^d^The gnomAD browser (https://gnomad.broadinstitute.org/).

^e^The Human Genetic Variation Database (http://www.hgvd.genome.med.kyoto-u.ac.jp/).

^f^Polymorphism Phenotyping v2 (http://genetics.bwh.harvard.edu/pph2/).

^g^Sorting Intolerant From Tolerant (http://sift.jcvi.org/).

^h^Combined Annotation Dependent Depletion (https://cadd.gs.washington.edu/).

^i^Mendelian Clinically Applicable Pathogenicity (http://bejerano.stanford.edu/mcap/).

^j^MutationTaster (http://www.mutationtaster.org/).

The p.Glu18Asp variant in patient 2 was found in dbSNP as rs779165187 (Table 1). Reportedly, its allele frequency in the general population was 0.21–0.26%. This variant was assessed as benign/tolerate by most of the five in silico programs, and has been submitted to the ClinVar database as a likely benign variant. Furthermore, the variant resided in exon 1, outside of the hotspot for BAMS-causative mutations^18^. Altogether, the p.Glu18Asp variant was classified as likely benign according to the ACMG/AMP guidelines. Thus, we excluded this variant from further analyses.

### Whole exome sequencing of patient 1

To exclude the possibility that CPHD of patient 1 was caused by a pathogenic variant in other genes, we performed exome sequencing for this individual. Consequently, we identified 64 heterozygous rare variants in 63 protein-coding genes, which were assessed as deleterious (Supplementary Table 1). However, none of the 63 genes, except for *SMCHD1*, was associated with IHH or CPHD.

### Clinical analysis of patient 1

We examined the clinical features of patient 1 with the p.Asp398Asn variant. This patient was a Japanese girl born to non-consanguineous parents at 37 weeks of gestation. Her birth weight was 2,216 g (− 1.3 SD). No dysmorphic features were noted. She had episodes of oxygen desaturation from four hours after birth, and was diagnosed with combined deficiencies of adrenocorticotropic hormone (ACTH), thyroid stimulating hormone (TSH), and growth hormone (GH). She received hydrocortisone and levothyroxine supplementation from the neonatal period, and GH treatment from eight months of age. She showed mild mental retardation from early childhood and underwent a special education program. She lacked spontaneous pubertal development in her teens and was diagnosed with HH. From 13 years of age, she received estrogen replacement therapy.

**Figure 2:**
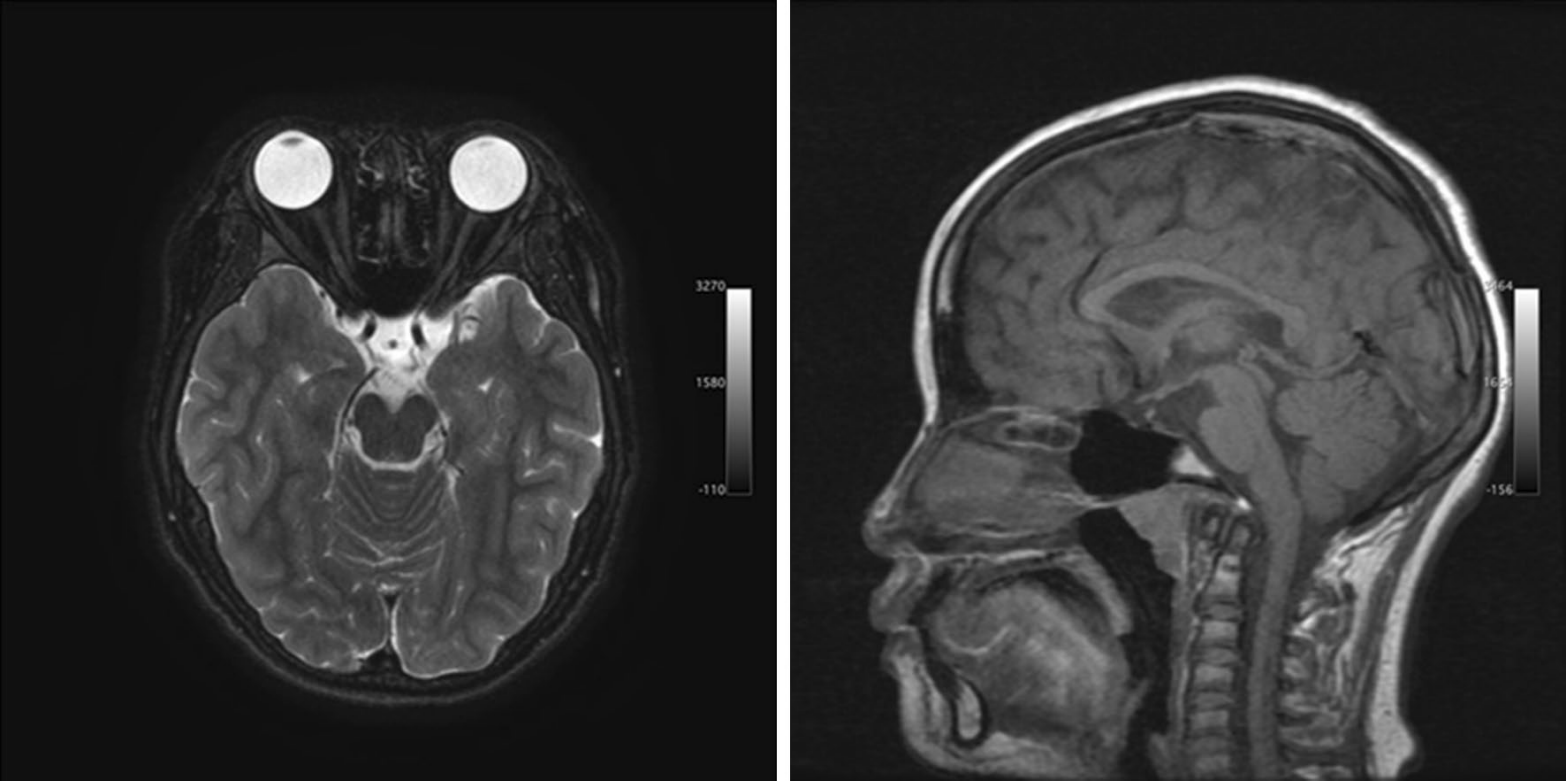
Brain magnetic resonance imaging of patient 1. Patient 1 exhibited ectopic posterior pituitary and optic nerve hypoplasia. The anterior pituitary lobe was not visible. The structures of the eyes and nose were apparently normal.

At 17 years of age, she measured 148 cm (− 1.9 SD) and weighted 60.2 kg (+ 1.0 SD). She retained normal visual activity with myopia. However, optical coherence tomography revealed decreased thickness of the retinal nerve fiber layer. Brain magnetic resonance imaging delineated ectopic posterior pituitary and optic nerve hypoplasia, together with normal eye and nose structure (Fig. 2). Endocrine evaluation demonstrated the lack of responses of ACTH (basal, < 0.2 pmol/L; stimulated, < 0.2 pmol/L), GH (basal, < 1.0 µg/L; stimulated, < 1.0 µg/L), and cortisol (basal, 8.3 nmol/L; stimulated, > 5.5 nmol/L) to the growth hormone releasing peptide 2 stimulation^25^. Thus, this patient satisfied the diagnostic criteria of SOD^23^. Physical evaluation detected no clinical features indicative of FSHD2^26,27^, except for mild fatigability, mild mental retardation, and slight instability in walking on tiptoes and heels (Supplementary Table 2).

The 55-year-old father, 51-year-old mother, and 23-year-old brother of patient 1 were phenotypically normal. Allegedly, the family had no history of congenital endocrine disorders, craniofacial anomalies, or muscular dystrophy.

### DNA methylation analysis

**Figure 3:**
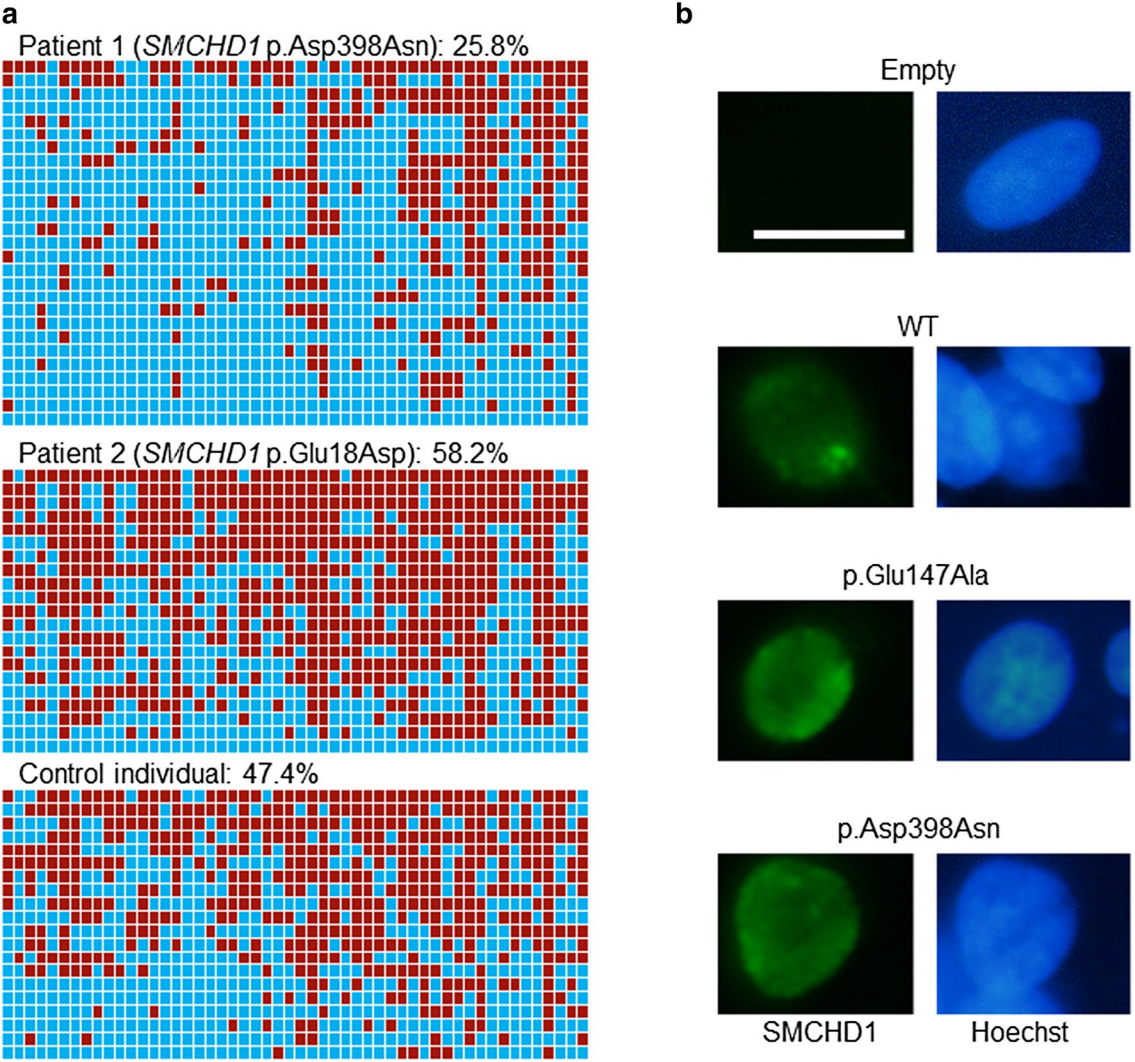
Functional analyses of the p.Asp398Asn variant. (**a**) The results of DNA methylation analysis. DNA methylation statuses of 52 CpG sites in the *DUX4* promoter region are shown^16^. The red and blue boxes depict methylated and unmethylated CpG sites, respectively. The average methylation rate of patient 1 was 25.8%, which is close to the cut off value of the diagnostic criteria of fascioscapulohumeral muscular dystrophy type 2 (25%)^19^. The average methylation rates of patient 2 and a control individual without SMCHD1 variants were 58.2% and 47.4%, respectively. (**b**) Representative results of in vitro functional assays. HEK293 cells transfected with the expression vectors of SMCHD1 or an empty vector were stained with anti-FLAG M2 for SMCHD1 (green) and Hoechst 33342 for DNA (blue). WT, full-length wildtype SMCHD1; p.Glu147Ala, an artificially created variant lacking the GHKL ATPase activity; p.Asp398Asn, a variant identified in patient 1. Scale bar, 20 μm.

We analyzed the DNA methylation status of patient 1 by bisulfite sequencing. Samples from patient 2 and a control individual without *SMCHD1* variants were also analyzed. The *DUX4* promoter region containing 52 SMCHD1-target CpG sites was PCR-amplified and subcloned^16,19^. We found that the average methylation rate of patient 1 was 25.8% (range of 27 clones, 0–80.8%) (Fig. 3a), which was markedly lower than previously reported data of control individuals (56.5%)^16^, and close to the cutoff value of the diagnostic criteria of FSHD2 (25%)^19^. Average methylation rates of patient 2 and the control individual were 58.2% and 47.4% respectively (Fig. 3a), which were almost comparable to the reference data of previous studies^16^.

Furthermore, we attempted to perform Southern blot analysis to examine the number of *D4Z4* repeats^28,29^. However, the quality and amount of the patient’s DNA sample were not sufficient for Southern blotting. Thus, it remains unknown whether patient 1 retains a normal number of *D4Z4* repeats.

### In vitro functional assays for the p.Asp398Asn variant

To analyze the effect of the p.Asp398Asn variant on protein function, we performed in vitro assays. It is known that wildtype (WT) SMCHD1 accumulates to the territory of the Xi, whereas an artificially created variant lacking GHKL ATPase activity (p.Glu147Ala) shows a diffuse distribution in the nucleus^14,15^. Thus, we transfected HEK293 cells with an expression vector for the p.Asp398Asn variant, together with expression vectors for the WT SMCHD1 and the p.Glu147Ala variant. As reported previously^14,15^, WT SMCHD1 was observed as nuclear foci (Fig. 3b). In contrast, both the p.Asp398Asn and p.Glu147Ala variants were diffusely distributed in the nucleus (Fig. 3b).

## Discussion

Mutation screening of *SMCHD1* in 74 patients with etiology-unknown IHH/CPHD identified two rare variants each in one patient. According to the ACMG/AMP guidelines, the p.Asp398Asn and p.Glu18Asp variants were classified into the likely pathogenic and likely benign groups, respectively. Several findings of this study support the pathogenicity of the p.Asp398Asn variant. First, this variant resides within the hotspot for BAMS mutations in an evolutionally constrained genomic region^18^, and has not been identified in the general population. Second, this variant was predicted to be damaging by all in silico programs used in this study. In particular, the high Combined Annotation Dependent Depletion (CADD) score of 26.2 is remarkable, because Shaw et al. have shown that *SMCHD1* variants with CADD scores of > 25.0 frequently result in BAMS^16^. Third, protein structural prediction suggested that this substitution alters hydrogen bonds. Specifically, it likely disrupts a hydrogen bond between the 398th and 400th residues, the latter being a nucleotide whose mutation results in BAMS^16^. Fourth, whole exome sequencing of patient 1 excluded other pathogenic variants in known IHH/CPHD-causative genes. Fifth, the relatively low DNA methylation rate of the SMCHD1-target CpG sites in patient 1 possibly reflects compromised activity of the mutant SMCHD1 protein as an epigenetic regulator^16,19^. Although the methylation rate was slightly above the cutoff value for the diagnostic criteria of FSHD2^19^, this does not argue against the pathogenicity of the p.Asp398Asn variant, because a certain percentage of previously reported BAMS patients with pathogenic *SMCHD1* mutations showed methylation rates higher than 30%^16^. Lastly, in vitro functional assays suggested functional impairment of the mutant SMCHD1. The p.Asp398Asn variant failed to accumulate in the Xi territory. The diffuse distribution pattern of the p.Asp398Asn variant was similar to that of an artificially created variant lacking GHKL ATPase activity^14^.

The phenotype of patient 1 with the p.Asp398Asn variant has both similarities and differences with that of previously reported BAMS patients. Patient 1 exhibited not only HH but also combined deficiencies of ACTH, TSH, and GH. On the other hand, she lacked ocular or nasal malformations, except for optic nerve hypoplasia and a thin retinal nerve fiber layer. These results indicate that the clinical consequences of *SMCHD1* mutations are broader than currently recognized. Actually, there seems to be a considerable overlap in the phenotype between the *SMCHD1* mutation-mediated syndrome and the HH-related disease spectrum, because septum pellucidum/corpus callosum hypoplasia, hearing loss, and cleft palate, HH, and ocular anomalies have been documented in both conditions^2–6^. However, considering that likely pathogenic *SMCHD1* variants were absent from 73 of 74 patients in our cohort, such variants appear to play only a minor role in the etiology of HH and its related disorders.

Previous studies have suggested that IHH in BAMS patients results from defective nasal placode formation and/or impaired projection of the gonadotropin releasing hormone neuron^16, 17^. However, such mechanism cannot account for the development of CPHD. Since SMCHD1 is known to regulate monoallelically expressed genes^11,13^, CPHD in our patient may reflect aberrant expression of some SMCHD1 target genes in the brain. Indeed, some genes involved in hypothalamus/pituitary development, such as *POU1F1* and *MKRN3*, were reported to show monoallelic expression^30,31^. Further studies are necessary to clarify the role of SMCHD1 in craniofacial and brain development.

Patient 1 exhibited no apparent clinical features of FSHD2. These results are consistent with previous findings that BAMS patients with *SMCHD1* mutations rarely manifest FSHD2^16^. The absence of muscle weakness in our patient and most BAMS patients may reflect the complex oligogenic or multifactorial nature of FSHD2^21^. Previous studies have suggested that *SMCHD1* mutations alone would not be sufficient to cause FSHD2^16,21^. Moreover, we cannot exclude the possibility that our patient may develop FSHD2 in later life^29^.

In summary, we identified two rare heterozygous *SMCHD1* variants in two of 74 patients with IHH and CPHD. Of the two variants, p.Asp398Asn was predicted to be a likely pathogenic variant. In vitro assay suggested functional impairment of the p.Asp398Asn protein. The results of this study provide the first indication that *SMCHD1* variants represent a rare genetic cause of the HH-related disease spectrum.

## Materials and methods

### Patients

The study group consisted of 31 patients clinically diagnosed with IHH and 43 patients with CPHD. Nineteen of the CPHD patients satisfied the diagnostic criteria of SOD, i.e., pituitary hormone deficiency associated with optic nerve hypoplasia and/or midline brain defects^23^. One patient with IHH showed eye anomaly (microphthalmia). Twenty-one of the IHH and one of the CPHD patients had anosmia, and were therefore diagnosed with Kallmann syndrome. None of the 74 patients had nasal malformations.

Prior to the present study, all participants underwent sequence analysis for 43 known causative genes of IHH and CPHD (Supplementary Methods). Patients carrying possibly pathogenic variants in these genes were excluded from this study.

### Mutation screening of *SMCHD1* for 74 patients with HH-related disorders

Mutation screening of *SMCHD1* was performed using genomic DNA samples of the patients. First, all 48 coding exons of *SMCHD1* and their flanking intronic regions were amplified using the HaloPlex HS Target Enrichment System (Design ID 31368-1548826336; Agilent Technologies, Palo Alto, CA, USA), and sequenced as 150 bp paired-end reads on a NextSeq sequencer (Illumina, San Diego, CA, USA). The methods for read alignment and variant call are described in the Supplementary Information. Then, we examined the position of detected variants in the SMCHD1 protein. Also, we referred to dbSNP (https://www.ncbi.nlm.nih.gov/snp/), the 1000 genome database (https://www.internationalgenome.org/1000-genomes-browsers/), the gnomAD browser (https://gnomad.broadinstitute.org/), and the Human Genetic Variation Database (http://www.hgvd.genome.med.kyoto-u.ac.jp/), to examine the frequency of variants in the general population. Polymorphisms that account for ≥ 1% of the alleles in the general population were excluded from further analysis. Next, we examined whether the variants have been submitted to the ClinVar database (https://www.ncbi.nlm.nih.gov/clinvar/).

Functional consequences of rare variants were predicted using five in silico programs, namely, Polymorphism Phenotyping v2 (PolyPhen-2, http://genetics.bwh.harvard.edu/pph2/), Sorting Intolerant From Tolerant (SIFT, http://sift.jcvi.org/), CADD (https://cadd.gs.washington.edu/), Mendelian Clinically Applicable Pathogenicity (M-CAP, http://bejerano.stanford.edu/mcap/), and MutationTaster (http://www.mutationtaster.org/). Variants with PolyPhen-2 scores of > 0.8, SIFT scores of < 0.05, CADD scores of > 20, M-CAP scores of > 0.025, or MutationTaster results of “disease causing” were assessed as damaging. The effects of the variants on the protein structure were predicted using PyMOL (version 2.3; Schrödinger). Lastly, the variants were classified according to the ACMG/AMP guideline for interpretation of sequence variants^24^. A variant scored as likely pathogenic was confirmed by Sanger sequencing. Primer sequences are available upon request.

### Whole exome sequencing for patient 1

Whole exome sequencing for patient 1 was performed in Macrogen Japan (Kyoto, Japan) using SureSelect Human All Exon Kit v6 (Agilent Technologies). We searched for rare variants in protein-coding genes, whose frequency in the general population is less than 1%. Next, we selected nonsense, frameshift, and splice-site mutations, as well as missense variants that were assessed as deleterious by all of the five in silico programs. Variants which were reported as benign or likely_benign in the ClinVar database were excluded. Lastly, we investigated whether the affected genes have previously been associated with IHH or CPHD^1,9^.

### DNA methylation analysis of patient 1

We examined DNA methylation of the 52 SMCHD1-target CpG sites in the *DUX4* promoter region at the *D4Z4* locus. We utilized the previously described methods with slight modifications^16,19^. In brief, the patient’s genomic DNA sample was treated with bisulfite using the EZ DNA Methylation-Gold Kit (ZYMO RESEARCH, Irvine, CA, USA). Then, the *DUX4* promoter region containing 52 CpG sites was PCR-amplified and subcloned into a TOPO TA vector (Invitrogen, Carlsbad, CA, USA). Twelve clones were picked and sequenced. The methylation rates of the region were calculated from the average of 20 or more clones. The results of the patient were compared to previous data obtained from *SMCHD1* mutation-negative control individuals^16,19^ and to the cutoff value for the diagnostic criteria of FSHD2 (25%)^19^.

### In vitro functional assay for the p.Asp398Asn variant

The expression vector for full-length WT SMCHD1 was created by inserting *SMCHD1* cDNA into a modified pcDNA5/FRT/TO vector (Invitrogen) with the FLAG-tag at the N-terminus^15^. Expression vectors for the p.Glu147Ala and p.Asp398Asn variants were created by site-directed mutagenesis. HEK293 cells were transiently transfected with WT or variant expression vectors or an empty vector using Lipofectamine 3000 (Invitrogen). After 48 h incubation, cells were fixed with 4% paraformaldehyde for 15 min, permeabilized with 0.25% Triton X-100 for 15 min, blocked with 3% fetal bovine serum blocking buffer, and incubated with Alexa 488-conjugated anti-FLAG M2 (F1804, Sigma-Aldrich, St. Louis, MO, USA) at room temperature for 1 h. Hoechst 33342 (Sigma-Aldrich) was used for nuclear staining. Fluorescence images were acquired using Olympus IX71 (Olympus cellSens software, Olympus Co., Tokyo, Japan). These experiments were repeated three times.

### Study approval

All methods were carried out in accordance with relevant guidelines and regulations. This study was approved by the Institutional Review Board Committee at the National Center for Child and Development and performed after obtaining informed consent from the participants or their parents.

## Supplementary information

Supplementary file1 (DOCX 27 kb)

Supplementary file2 (XLSX 21 kb)

Supplementary file3 (XLSX 11 kb)

## Data Availability

The datasets generated during and/or analyzed during the current study are available from the corresponding author on reasonable request.
