# Supplementary material for: Rare variant of the epigenetic regulator SMCHD1 in a patient with pituitary hormone deficiency
Source: Sci Rep. 2020 Jul 3;10:10985. doi: 10.1038/s41598-020-67715-x (PMC7335161; doi:10.1038/s41598-020-67715-x)
Supplement: Supplementary file 1 — Supplementary file1 (DOCX 27 kb) [file 41598_2020_67715_MOESM1_ESM.docx]

**Supplementary Methods**

**Rare variant of the epigenetic regulator SMCHD1 in a patient with pituitary hormone deficiency**

Kenichi Kinjo^1,2^, Keisuke Nagasaki^3^, Koji Muroya^4^, Erina Suzuki^1^, Keisuke Ishiwata^5^, Kazuhiko Nakabayashi^5^, Atsushi Hattori^1,6^, Koji Nagao^7^, Ryu-Suke Nozawa^7^, Chikashi Obuse^7^, Kenji Miyado^8^, Tsutomu Ogata^2^, Maki Fukami^1^ & Mami Miyado^1^

^1^Department of Molecular Endocrinology, National Research Institute for Child Health and Development, Tokyo, Japan

^2^Department of Pediatrics, Hamamatsu University School of Medicine, Hamamatsu, Japan

^3^Department of Homeostatic Regulation and Development, Niigata University Graduate School of Medical and Dental Sciences, Niigata, Japan

^4^Department of Endocrinology and Metabolism, Kanagawa Children's Medical Center, Yokohama, Japan

^5^Department of Maternal-Fetal Biology, National Research Institute for Child Health and Development, Tokyo, Japan

^6^Department of Advanced Pediatric Medicine, Tohoku University School of Medicine, Tokyo, Japan

^7^Department of Biological Sciences, Graduate School of Science, Osaka University, Osaka, Japan

^8^Department of Reproductive Biology, National Research Institute for Child Health and Development, Tokyo, Japan

**Mutation screening of known causative genes for isolated hypogonadotropic hypogonadism and combined pituitary hormone deficiency**

We performed next-generation sequencing (NGS) for 43 known causative genes for isolated hypogonadotropic hypogonadism (IHH) and combined pituitary hormone deficiency (CPHD). These genes consisted of 25 causative genes for IHH (*ANOS1, CCDC141, DUSP6, FGF17, FLRT3, FSHB, GNRH1, GNRHR, HS6ST1, IL17RD, KISS1, KISS1R, KLB, LEP, LEPR, LHB, NELF, NR0B1, PROK2, RNF216, SEMA3A, SOX10, SPRY4, TAC3,* and *TACR3*), nine genes for CPHD (*BMP4, GLI2, IGSF1, LHX4, OTX2, PAX6, POU1F1, SHH,* and *SOX3*), and nine genes for both conditions (*CHD7, FGF8, FGFR1, HESX1, LHX3, PROKR2, PROP1, SOX2,* and *WDR11*) [1, 2].

Genomic DNA samples were obtained from peripheral leukocytes. The coding regions of the abovementioned genes were amplified using the HaloPlex HS Target Enrichment System (Design ID 31368-1548826336; Agilent Technologies, Palo Alto, CA, USA), according to manufacturer’s instructions. The library was sequenced as 150 bp paired-end reads on a NextSeq sequencer (Illumina, San Diego, CA, USA).

**NGS data analysis**

Base calling, read filtering, and demultiplexing were performed with a standard Illumina pipeline. We used BWA 0.7.17 (https://sourceforge.net/projects/bio-bwa/files) with default settings to map sequence reads against the human genome. Local realignment, quality score recalibration, and variant call were performed with GATK3.8 [3]. ANNOVAR [4] was used to annotate called variants.

**Interpretation of *SMCHD1* variants**

Rare *SMCHD1* variants were classified according to the American College of Medical Genetics and Genomics/Association for Molecular Pathology (ACMG/AMP) guideline for interpretation of sequence variants [5]. Of the two variants, the p.Asp398Asn variant was assessed likely pathogenic, because this variant satisfied two criteria of moderate evidence of pathogenicity, *i.e*., located in a mutational hot spot (PM1) and absent from databases (PM2), as well as two criteria of supporting evidence, *i.e*., a missense variant in a gene that has a low rate of benign missense variation and in which missense variants are a common mechanism of disease (PP2) and predicted to be damaging by multiple computational programs (PP3). The p.Glu18Asp variant was scored as likely benign, because this variant satisfied two criteria of supporting evidence of benign impact, *i.e*., assessed as benign by multiple computational programs (BP4) and has been registered as likely-benign in the ClinVar database (BP6).

**References**

1. Topaloğlu, A. K. Update on the genetics of idiopathic hypogonadotropic hypogonadism. *J. Clin. Res. Pediatr. Endocrinol.* **9 (Suppl 2)**, 113–122 (2017)

2. Fang, Q. et al. Genetics of combined pituitary hormone deficiency: roadmap into the genome era. *Endocr. Rev.* **37**, 636–675 (2016)

3. McKenna, A. et al. The Genome Analysis Toolkit: a MapReduce framework for analyzing next-generation DNA sequencing data. *Genome. Res.* **20**, 1297–1303 (2010)

4. Wang, K., Li, M. & Hakonarson, H. ANNOVAR: functional annotation of genetic variants from high-throughput sequencing data. *Nucleic. Acids. Res.* **38**, e164 (2010)

5. Richards, S. et al. Standards and guidelines for the interpretation of sequence variants: a joint consensus recommendation of the American College of Medical Genetics and Genomics and the Association for Molecular Pathology. *Genet. Med*. **17**, 405–424 (2015)
